# A landscape of mouse mitochondrial small non-coding RNAs

**DOI:** 10.1371/journal.pone.0293644

**Published:** 2024-01-02

**Authors:** Chiara Siniscalchi, Armando Di Palo, Giuseppe Petito, Rosalba Senese, Francesco Manfrevola, Ilenia De Leo, Nicola Mosca, Teresa Chioccarelli, Veronica Porreca, Giovanna Marchese, Maria Ravo, Rosanna Chianese, Gilda Cobellis, Antonia Lanni, Aniello Russo, Nicoletta Potenza

**Affiliations:** 1 Department of Environmental, Biological, Pharmaceutical Sciences and Technologies, University of Campania “Luigi Vanvitelli”, Caserta, Italy; 2 Department of Experimental Medicine, University of Campania “Luigi Vanvitelli”, Naples, Italy; 3 Genomix4Life S.r.l., Baronissi (SA), Italy; 4 Genome Research Center for Health, CRGS, Baronissi, Italy; Institute of Parasitology and Biomedicine, SPAIN

## Abstract

Small non-coding RNAs (ncRNAs), particularly miRNAs, play key roles in a plethora of biological processes both in health and disease. Although largely operative in the cytoplasm, emerging data indicate their shuttling in different subcellular compartments. Given the central role of mitochondria in cellular homeostasis, here we systematically profiled their small ncRNAs content across mouse tissues that largely rely on mitochondria functioning. The ubiquitous presence of piRNAs in mitochondria (mitopiRNA) of somatic tissues is reported for the first time, supporting the idea of a strong and general connection between mitochondria biology and piRNA pathways. Then, we found groups of tissue-shared and tissue-specific mitochondrial miRNAs (mitomiRs), potentially related to the “basic” or “cell context dependent” biology of mitochondria. Overall, this large data platform will be useful to deepen the knowledge about small ncRNAs processing and their governed regulatory networks contributing to mitochondria functions.

## Introduction

The recent advances in high-throughput sequencing technologies shed light on a plethora of RNA species with limited coding potential, collectively called non-coding RNAs (ncRNA) [1]. Besides ribosomal RNA, ncRNA molecules are generally classified according to their size threshold in long ncRNA (lncRNAs), longer than 200 nt up to several kilobases, and small or short ncRNAs, from few to 200 nt. lncRNA are involved in the regulation of gene expression at transcriptional and post-transcriptional levels by interacting with DNA, proteins and other RNA molecules. Short ncRNAs include the well known tRNA (transfer RNA), involved in the translation of mRNA; snRNA (small nuclear RNA), involved in splicing; snoRNA (small nucleolar RNA), implicated in ribosomal RNA modification; Piwi-interacting RNA (piRNA) and microRNAs (miRNA) [2].

piRNAs, 24–35 nucleotide long, 2’-O-methyl modified at 3’-end, associate with PIWI proteins; they were discovered in germlines in 2001, representing the most abundant small RNAs in mammalian testes and are essential for fertility; they are classically associated with “genome defense” against transposable elements and viruses [3–5]; more recently, they have been involved in epigenetic modification and gene expression regulation, probably at lower extent also in somatic tissues [6]. piRNA biogenesis and functioning are still not completely known, probably due to the occurrence of different production pathways, the wide diversity of functions (in the nucleus and cytosol) and poorly conserved pathways specifically associated to features of germ cell development [6, 7].

miRNAs, the most studied group of small ncRNAs, with a size of about 20 nt, negatively regulate gene expression by binding to target transcripts, thus affecting their translation and/or stability [8]. In mammals, up to 50% of all protein-coding transcripts are predicted to be targeted by miRNAs; in addition, one miRNA can bind different mRNAs and one mRNA can targeted by different miRNAs, giving rise to complex regulatory networks that play key roles in almost all physiological pathways, but also in the pathogenesis of several diseases, especially cancer [9–11]. miRNAs biogenesis is compartmentalized between nucleus and cytoplasm, but they are largely operative in the cytoplasm. However, emergent data indicate that mature miRNAs can localize in the nucleus and in subcellular compartments in the cytoplasm, unveiling possible new and specialized functions related to specific cellular compartments [12]. In this regard, some studies have detected microRNAs in mitochondria isolated from rat, mouse and human cells, collectively called mitomiRs (mitochondria-localized microRNA) [13, 14]. In fact, in 2006 a few miRNAs were found in rat’s liver mitochondria; although initially thought as cytosolic contamination, few years later other studies performed by microarray profiling and more recently by RNAseq confirmed the localization of miRNAs in mitochondria [15–22].

Mitochondria are the powerhouses in the cell and essential organelles for the integration of several key metabolic processes such as respiration, fatty and amino acid metabolism [23]. They can be defined as central regulators of cellular homeostasis, and dysregulation of any activity has pathological consequences, like cancer, inflammation, neurodegeneration [24–27]. Mitochondria contain their own circular genome, about 16 kbp in length, with 37 genes producing 2 rRNAs, 22 tRNAs and 13 proteins of the oxidative phosphorylation (OXPHOS) system [28]. Mitochondrial homeostasis is dependent on the expression of its own genes, quite limited in number, but largely on the import of nuclear-encoded proteins from the cytoplasm [13]. It is becoming increasingly clear that nuclear genome contributes to mitochondria activities not only through proteins (about 1500 nuclear-encoded proteins), but also through a number of miRNAs that can affect transcripts encoding mitochondrial-localized and mitochondrial-related proteins. This crosstalk between nucleus and mitochondria is essential for regulating mitochondria dynamics (biogenesis, fusion, fission, mitophagy), metabolic pathways and mitochondria-mediated apoptosis, whose dysregulations have pathological consequences. Indeed, some miRNAs can be translocated to mammalian mitochondria. The proposed import mechanisms include a detachment of AGO2 and miRNA from RISC or just GW182 due to AGO2 phosphorylation or some other signal activation promoting their translocation together or separately; this process could be stimulated by P-bodies; translocation across mitochondrial membranes is unknown but suggested to be promoted by PNPase and occurs within TOM/TIM complexes, or relies on VDAC at the outer mitochondrial membrane [13, 29, 30].

Different gaps in the knowledge emerge from literature related to ncRNA contribution to mitochondria biology. As an example, mitomiR function still remains controversial, especially due to debated presence of RISC components required for their canonical mechanism of silencing activity [12, 31]. Moreover, a deep sequencing analyses of small RNA content in mitochondria, probably a foundation for a systematic understanding, is available only for HEK293 and HeLa human cells, and human myotubes [20, 22], and regarding to mouse model only for primordial male germ cells, spermatogonia, spermatozoa, oocytes and zygotes [21]. To gain insights into possible new and specialized function of small RNAs and in particular miRNAs related to mitochondrial biology, this work explores the content of small RNAs population by RNAseq of mitochondria isolated from different mouse tissues, also in order to identify a possible shared pool and distinctive mitomiR signatures potentially involved in basic or cell-context dependent mitochondria functions.

## Materials and methods

### Animals and ethics statement

Three wild-type male CD1 mice, ∼3 months old, were used in this study. They were maintained in a temperature-controlled room at 22 ± 2°C under a 12 h dark/light cycle with a standard pellet diet and unlimited access to water.

Mice were sacrificed under anesthesia by cervical dislocation. In detail, animals were placed in a plexiglas chamber with 4% isoflurane (Iso-Vet, Piramal Healthcare, United Kingdom) for 5 min and were sacrificed when fully sedated, as measured by a lack of heartbeat and active paw reflex. Tissues were rapidly removed, rinsed in PBS (pH 7.6) to remove blood contaminants and used for experimental procedures. Every effort was made to minimize animal pain and suffering.

Experiments were approved by the Italian Ministry of Education and the Italian Ministry of Health (authorization n°48/2022-PR issued on 28.01.2022). Procedure involving animal care were carried out in accordance with the National Research Council’s publication Guide for Care and Use of Laboratory Animals (National of Institutes of Health Guide).

### Mitochondria purification

Mitochondria were purified from freshly excised tissues (liver, gastrocnemius skeletal muscle, testis, white adipose tissue) using Qproteome Mitochondrial Isolation kit (Qiagen) according to manufacturer’s instructions with minor modifications and performing all the steps at 4°C. Briefly, tissues were homogenized in Lysis Buffer supplemented with Protease Inhibitor solution by Potter homogenizer, incubated on an end-over-end shaker for 10 min and centrifuged at 1000 x g for 10 min. The pellet was resuspended in ice-cold Disruption Buffer supplemented with Protease Inhibitor, subjected to Potter homogenizer for cell disruption, followed by centrifugation at 1000 x g for 10 min. The supernatant was centrifuged at 6000 x g for 10 min to pellet down mitochondria. The mitochondria were further purified by resuspending the pellet in Purification buffer and carefully pipetting onto layers of Purification Buffer and Disruption Buffer. The solution was centrifuged at 14000 x g for 15 min. The mitochondrial ring at the interface of Purification Buffer/Disruption Buffer was collected and washed in Mitochondria Storage Buffer; the final mitochondrial pellet was resuspended in 20 μl Storage buffer.

In order to remove RNA of nuclear origin present in the cytosol and eventually adsorbed on the outer mitochondrial membrane, mitochondria were treated with RNase A solution, as already described [20, 22]. In brief, the 20 μl purified mitochondria from the above described procedure were mixed with 300 μl PBS containing RNase A (100 μg/ml) and incubated at 37°C for 1 h. Rnase A treated mithocondria were pelleted down at 8000 rpm for 10 min, washed twice in PBS and finally resuspended in Qiazol buffer (Qiagen) for RNA extraction.

### RNA extraction, control of mitochondria purity and real-time PCR analyses

Mitochondria and cytosolic fractions obtained by the procedure described above according to Qproteome Mitochondrial Isolation kit (Qiagen) protocol were used for total RNA purification by miRNeasy Mini Kit (Qiagen) according to the manufacturer’s protocol. The yield and quality of RNA were evaluated by NanoDrop One spectrophotometer (NanoDrop), Qubit 4 Fluorometer (Invitrogen) and by TapeStation 4200 (Agilent Technologies).

The mitochondrial RNA enrichment and eventual cytosolic RNA contamination were checked by RT-qPCR. Mitochondria and cytosolic RNAs were retrotranscribed by SensiFAST cDNA Synthesis kit (Bioline). Then standard SYBR Green Real-time qPCR assays were performed for amplifying β-actin transcript as nucleus-derived RNA control and Cytochrome c as mitochondrion-derived control with the following primers: β-actin, 5’-CAACGGCTCCGGCAT -3’ and 5’- CTCTTGCTCTGGGCC -3’; Cytochrome b, 5′-TACCTGCCCCATCCAACATT-3′ and 5′-TAAGCCTCGTCCGACATGAA-3′. For the further experimental steps, we selected the RNA mitochondrial preparations giving the following qPCR parameters on triplicate analyses: Ct>35 for β-actin, which was fully detectable in the parallel cytosolic sample, indicating the absence of cytosolic contaminant RNA; Ct<28 for cytochrome b, indicating a good enrichment of mitochondrial RNA.

miR-122-5p, miR-133a-3p, miR-29a-3p, miR-15b-5p were quantified along with U6 (reference transcript) by RT-qPCR with TaqMan^®^ miRNA assays from Applied Biosystems according to the manufacturer’s protocol. The expression levels of miRNAs were normalized to the reference transcript by using the 2^-ΔCt^ method.

### RNA sequencing and data analyses

Indexed libraries were prepared from 50 ng of each purified RNA sample with NEXSmallRNA Seq v3.0 (PerkinElmer) according to the manufacturer’s instructions. Libraries were quantified using the TapeStation 4200 (Agilent Technologies) and Qubit 4 Fluorometer (Invitrogen). Libraries were pooled in equimolar amounts with final concentration of 2 nM. The pooled samples were subject to cluster generation and sequencing using an Illumina NextSeq550Dx System (Illumina Inc.) in a 1x75 single-end format with an average depth of the sequences of 20 Million reads/sample. The raw sequence files generated (.fastq files) underwent quality control analysis using FastQC (http://www.bioinformatics.babraham.ac.uk/projects/fastqc/). sRNAbench by sRNAtoolBox was used to identify the non-coding RNAs signature in all sequenced samples, thus removing adapter sequences, based on reference kit instructions, and the low quality reads [32]. In particular, the following steps were performed: 4 nt barcode from 5’ end of reads were removed; the adapter sequence "TGGAATTCTCGGGTGCCAAGGG" were detected; minimum length of adapter sequence aligned considered as 10 and only 1 mismatch allowed between adapter and reference sequences; 4 nt random adapter from 3’ end of adapter trimmed reads were removed.

The samples were mapped respect to miRBase database (version22 –GRCh38 ‐ GCA_000001405.15) to Ensembl and to RNACentral annotatio, to obtain several classes of ncRNAs allowing only 1 mismatch. piRNA-related reads were also mapped by Bowtie2 on the IPpiRNAdb (https://github.com/OdBT/piRNA-IPdb_v2) [33] obtaining an overall alignment rate equal to 99.05% for gastrocnemius skeletal muscle samples, 98.81% for liver samples, 99.32% for testis samples, 98.85% for WAT samples.

Finally, reads were also aligned to mouse mitochondrial genome (NC_005089.1 from NCBI) by blastn command line applications with defaults parameters, finding reads with at least one reported alignment in the following percentages: 4.87% for gastrocnemius muscle, 6.17% for liver, 4.00% for testis and 3,47% for WAT. e-value≤0.01 was considered statistically significant. Finally, all piRNA sequences of piRbase (http://bigdata.ibp.ac.cn/piRBase/) and all miRNA sequences included in miRbase (https://www.mirbase.org/) were aligned to mitochondrial genome with parameters described above.

Sequencing data are available in the ArrayExpress database repository (https://www.ebi.ac.uk/arrayexpress/) with the following accession number: E-MTAB-12911.

### mitomiR targetome analyses

The list of 126 tissue-shared mitomiRs and the lists of tissue-specific mitomiRs were used to launch a search on miRWalk platform (http://mirwalk.umm.uni-heidelberg.de/) to retrieve all their target transcripts. In detail, by selecting “miRTarbase”, or “TargetScan” and “miRDB”, and “5′UTR”, or “3′ UTR” or “coding region” and selecting 0.5 as score for miRNA–mRNA pairings, 6 different target lists for tissue-shared and the 4 tissue-specific mitomiRs subset were retrieved containing all experimentally validated targets (by miRTarbase tool), or those predicted by both TargetScan and miRDB tools. By exporting the lists in Excel, a list of non-redundant transcripts representing the whole targetome of tissue-shared and 4 lists of tissue-specific mitomiRs were produced. On those lists, enrichment of biological pathways supplied by Kyoto Encyclopedia of Genes and Genomes (KEGG) pathway annotation was performed by Database for Annotation, Visualization and Integrated Discovery (DAVID, https://david.ncifcrf.gov) which facilitates the transition from data collection to biological analysis by systematically extracting biological meaning and gene functional groups from large gene lists. Considering this type of analysis could increase the false-positive rate, to control the false-positive rate in the results, a multiple test correction of enrichment p values was performed on the functional annotation categories by selecting the Benjamini–Hochberg procedure. Biological pathways with *p* values < 0.05 were considered statistically significant [34].

Targetomes of tissue-shared or–specific mitomiRs were also analyzed by MitoCarta3.0 to extract enriched biological pathways specifically related to mithocondria biology. In brief, targetome lists were matched with 1140 mouse genes encoding proteins with strong support of mitochondrial localization; the merging target transcript lists, plus those mitochondrial transcripts reported in PubMed as experimentally validated target of our detected mitomiRs, were analyzed by MitoCarta3.0 for the assignment to 149 hierarchical ’MitoPathways’ spanning seven broad functional categories relevant to mitochondria (http://www.broadinstitute.org/mitocarta) [35].

## Results

### Rationale of the study

**Fig 1:**
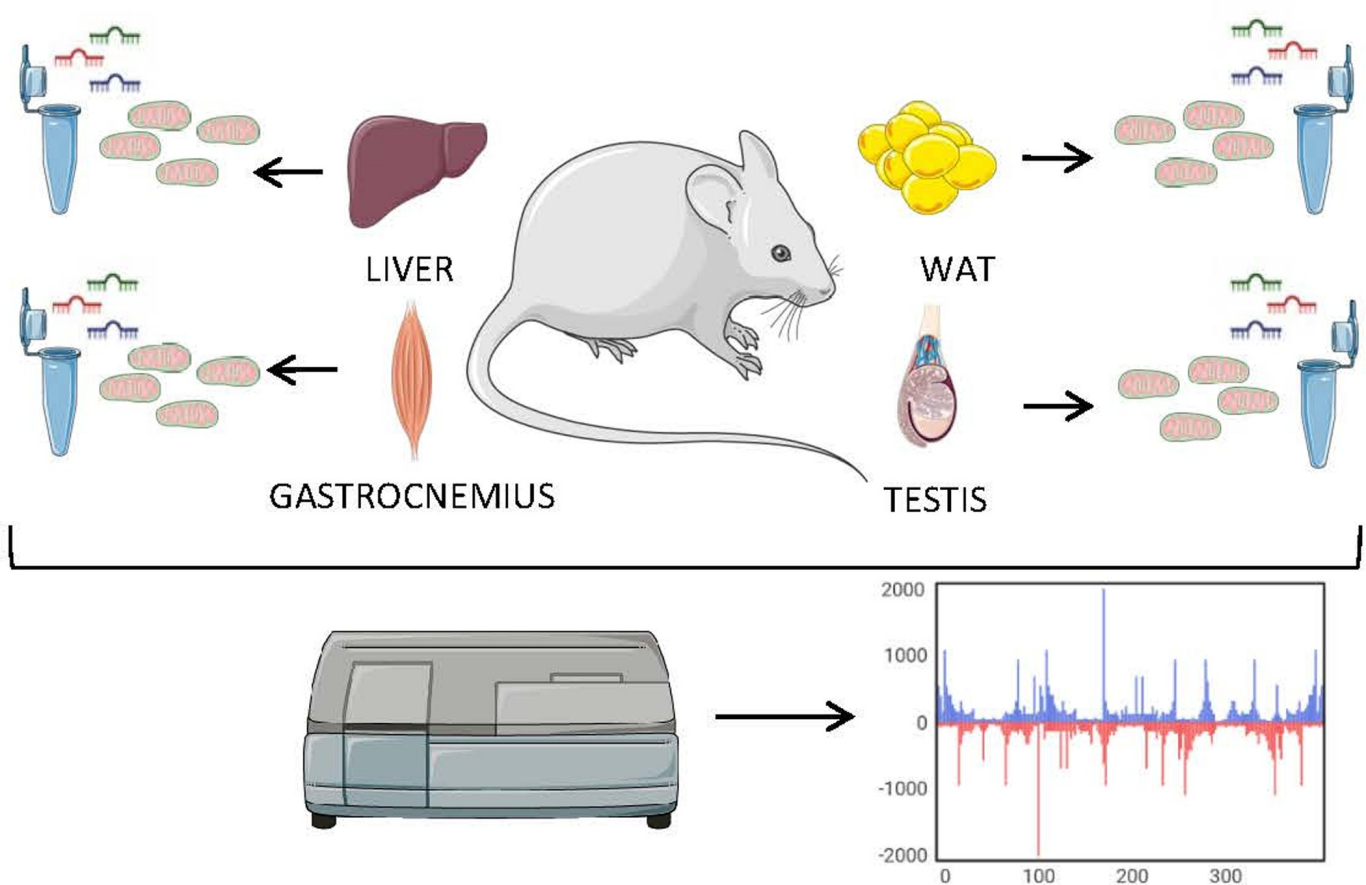
Rationale of the study. Illustration created with Servier Medical Art, provided by Servier, licensed under a Creative Commons Attribution 3.0 Unported license.

In order to have a large platform of data regarding the population of small RNAs in mitochondria, arguing their potential contribution to mitochondrial activities, novel mechanisms of regulation and crosstalk with other cellular components, we decided to select (Fig 1):

- liver, since it can be seen as the metabolic hub of the body, in analogy to mitochondria being the metabolic hub of the cell; in fact, the liver is extremely rich in mitochondria, with each hepatocyte containing 1000–2000 mitochondria [36];
- gastrocnemius skeletal muscle, whose health is dependent on the optimal function of its mitochondria [37]; due to the high metabolic requirements for locomotion, mitochondrial ATP production is very important, with typically 3–8% of the skeletal muscle volume being mitochondria [38];
- testis, since spermatogenesis relies on mitochondrial dynamics and functions, whose disruption causes male infertility [39];
- white adipose tissue (WAT), where mitochondrial activity is essential for the choice of carbon source (use of carbohydrate through glycolysis versus use of lipids for oxidative phosphorylation) and impacts on tissue and systemic energy homeostasis; the important role of mitochondria in the regulation of white adipose tissue remodeling and energy balance is increasingly appreciated [40].

**Fig 2:**
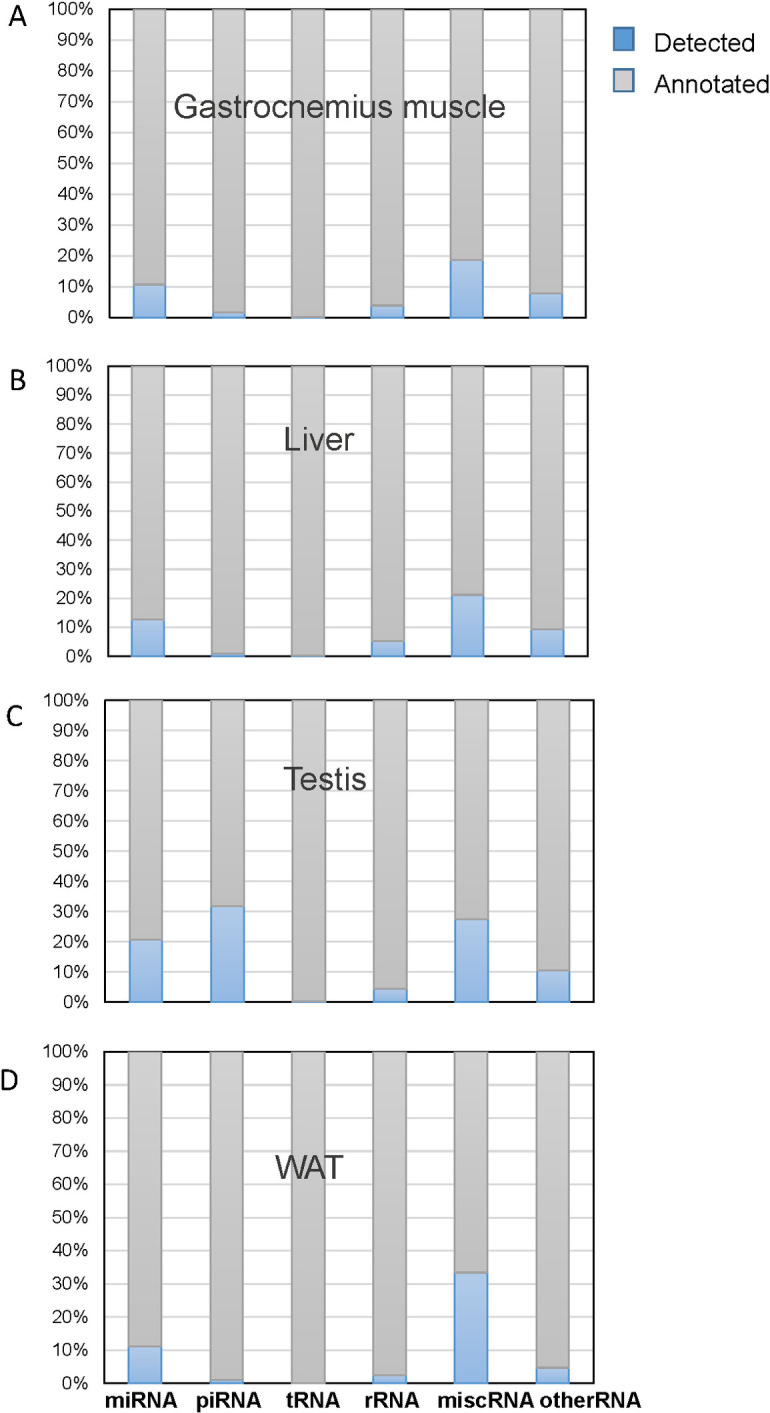
ncRNAs identified in the current study. The light-blue indicates RNA biotypes percentages detected in indicated mitochondria tissue on the annotated ones in the different databases, i.e. RNA-Central, Ensembl (GRCm38_p5) and miRBase (v22). Misc_RNA: miscellaneous RNA; otherRNA: snRNA, snoRNA and RNA fragments are included.

Mitochondria were purified as described in Material and Methods section, and smallRNA sequencing experiments were performed on extracted RNAs (Fig 1). The percentage of detected mitochondrial smallRNA populations on the annotated ones for each tissue are reported in Fig 2 and detailed below.

### mitopiRNAs

**Fig 3:**
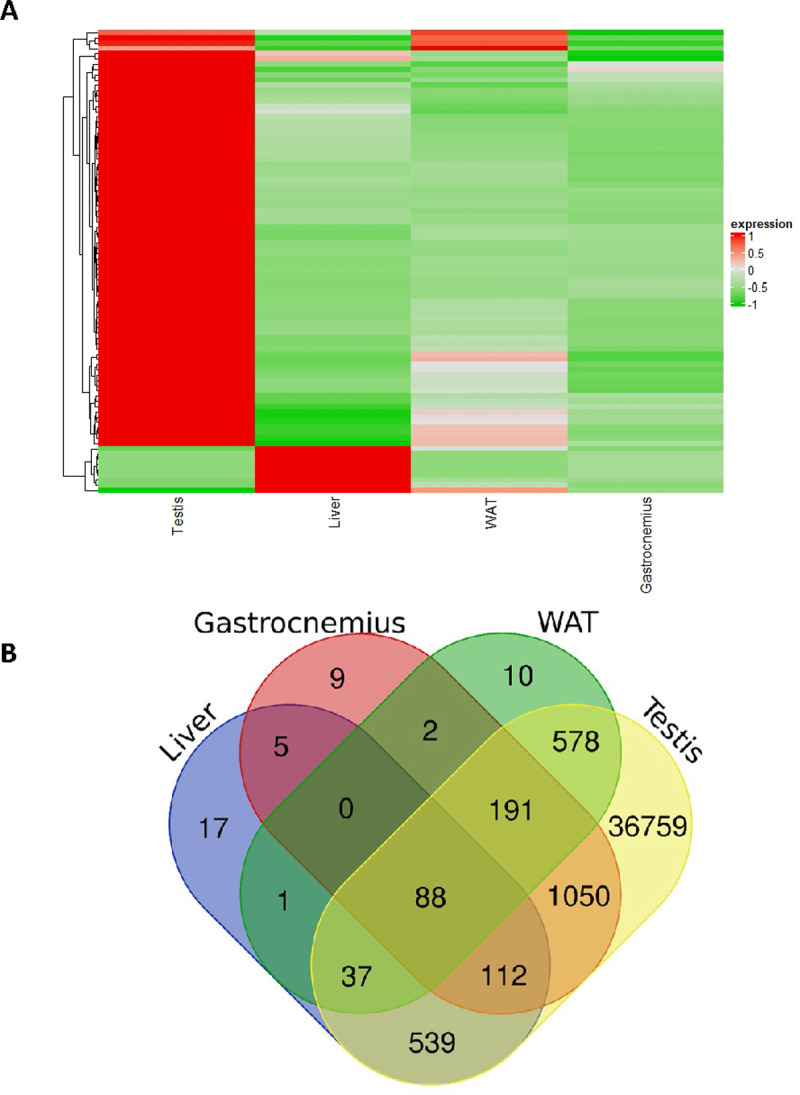
mitopiRNAs expression analyses. (A) Normalized expression unsupervised heatmap relative to piRNAs in the mitochondria of the indicated tissues, obtained by using the software ComplexHeatmap, a package of R. (B) The Venn diagram displaying the numbers of overlapping mitopiRNAs (Raw Count Mean ≥1) among the different tissues.

piRNAs are largely present in the mitochondria of testis, representing the most predominant population of small RNAs (Fig 2); their finding into mitochondria of testis is consistent with bioinformatic analyses on smallRNAseq libraries from primordial germ cells and spermatozoa of male mice [21, 41]. Surprisingly, here piRNAs were also detected in the mitochondria of the other tissues, representing a novelty in the current knowledge (S1 Table). In fact, although their presence has been already described for different somatic tissues [42] and related to epigenetic reprogramming and gene expression regulation [3, 43], piRNAs specifically located in mitochondria are only reported in mammalian cancer cells [22, 44] and inferred by bioinformatic analyses on RNA extracted from the whole cell lysates derived from murine primordial germ cells, spermatozoa oocytes, zygotes and embryonic gonadal cells [21, 41]. Two studies have demonstrated a role of the mitochondrial protein MitoPLD/Zucchini (mammalian/Drosophila homologues) in piRNA biogenesis and piRNA mediated silencing in mouse and fly germlines [45, 46]; finally, PIWIL-1, a human homologue of the mouse MIWI protein, has been detected in the mitochondria of human cells [44] pointing for a mitochondrial role in the biogenesis of piRNAs. Our discovery of piRNA populations in mitochondria of somatic tissues strongly supports the idea that the organelles may be an important and general player in piRNA pathways, beyond germ line tissues. Intriguingly, their expression pattern appears different for analyzed tissues, showing their massive presence in testis and some overlapping among analyzed tissues (Fig 3). Analogously to the so-called mitomiR, those piRNAs can be indicated as mitopiRNAs.

### mitomiRNAs

The RNAseq analyses revealed similar percentages of miRNAs on the annotated ones in the mitochondria of gastrocnemius muscle, liver and WAT and a higher content in testis (Fig 2).

**Fig 4:**
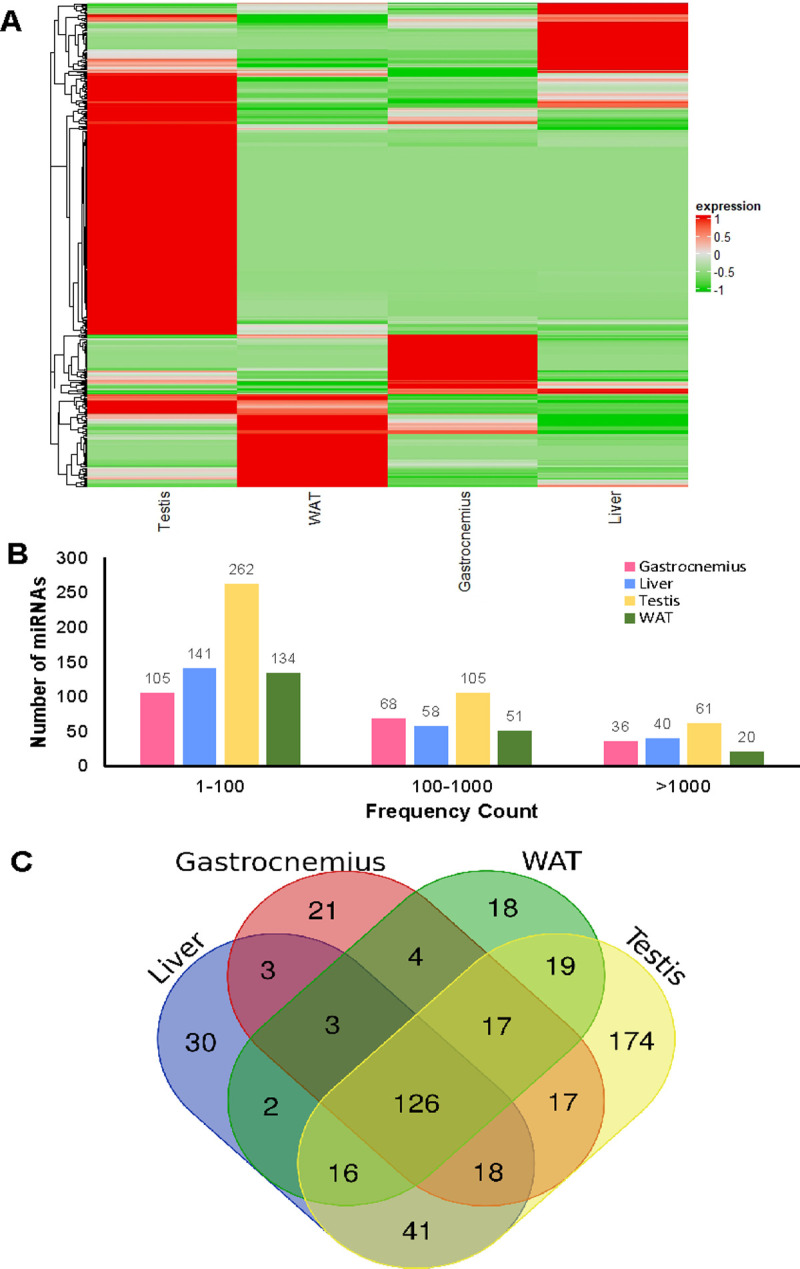
mitomiRNAs expression analyses. (A) Normalized expression unsupervised heatmaps relative to mitomiRs of the indicated tissues, obtained by using the software ComplexHeatmap, a package of R. (B) Distribution of mitomiR levels with respect to frequency; numbers of sequence reads (showed on the bars) were taken as miRNA levels and the values are represented in the form of range of values in mitochondrial sRNA libraries of each tissue. (C) The Venn diagram displaying the numbers of overlapping mitomiRs among the different tissues.

The overall expression pattern of mitomiRs is distinctive for the different tissues and ranges from few read counts to more than 1000, whose frequency counts is smaller compared to others (Fig 4A and 4B). We hypothesized that some miRNAs found in the mitochondria could be related to the basic functions of the organelle, whereas others could be mainly associated to specialized functions depending on the tissue. To explore that hypothesis, all the miRNAs found in the mitochondria of each tissue, from very low (> 1) to higher read counts were analyzed, resulting in a core of 126 shared mitomiRs, some others overlapping among the tissues and a number of tissue-specific mitomiRs (Fig 4C and S2 Table). Different papers and related datasets consistently report their expression also in tissues [47–49], and for some of them targets related to mitochondria functioning have been reported (Tables 1–5), thus further connecting their relevance for mitochondria biology.

**Table 1:** Tissues-shared top-10 mitomiRs according to their reads count.

miRNA name	chromosome location	reads count*	Validated target related to mitochondria functioning [reference]
mmu-miR-122-5p	chr 18	158456.4	Hand2, involved in mitochondrial fission [50] Arl-2, involved in energy homeostasis [51]
mmu-miR-1a-3p	chr 2	151875.1	Nd1 and Cox1, involved in OXPHOS [52]
mmu-let-7f-5p	chr 13	18780.3	IL-23R, related to mitochondrial respiration [53] ATP5B, involved in OXPHOS [54]
mmu-let-7a-5p	chr 13	16561.3	Acsl6, related to mitochondria dysfunction [55] Nd4, involved in OXPHOS [56]
mmu-let-7c-5p	chr 16	15125.1	nd
mmu-miR-143-3p	chr 18	13428.5	Bcl-2, involved in mitochondria- mediated apoptosis [57] FGF21, related to mitochondrial dysfunction [58]
mmu-miR-26a-5p	chr 9	11590.5	Cox5a, involved in OXPHOS [59] Creb1, Stk4, related to mitochondria dysfunction [60]
mmu-let-7g-5p	chr 9	9859.1	nd
mmu-let-7b-5p	chr 15	9291.6	PGC1alfa, related to mitochondrial respiration [61] Cytochrome b, IRS1, related to mitochondria-mediated ROS production and lipid deposition [62]
mmu-miR-133a-3p	chr 18	6882.9	IGF1R, related to mitochondrial stress [63] Dio3, involved in mitochondrial dysfunction [64]

*Reads count mean from the different tissues

nd, not yet determined

**Table 2:** Gastrocnemius-specific top-10 mitomiRs according to their reads count.

miRNA name	chromosome location	reads count	Validated target related to mitochondria functioning [reference]
mmu-miR-133b-3p	chr 1	160.4	nd
mmu-miR-365-3p	chr 16	102.4	nd
mmu-miR-615-3p	chr 15	89.0	nd
mmu-miR-487b-3p	chr 12	81.2	nd
mmu-miR-133a-5p	chr 18	72.6	Prdm16, related to mitochondria-mediated thermogenic activity [65]
mmu-miR-127-5p	chr 12	48.9	β-F1-ATPase, involved in OXPHOS [66]
mmu-miR-5110	chr 11	45.0	nd
mmu-miR-6946-5p	chr 14	39.0	nd
mmu-miR-345-3p	chr 12	34.6	nd
mmu-miR-1964-3p	chr 7	28.4	nd

nd, not yet determined

**Table 3:** Liver-specific top-10 mitomiRs according to their reads count.

miRNA name	chromosome location	reads count	Validated target related to mitochondria functioning [refernce]
mmu-miR-3068-3p	chr 12	341.0	nd
mmu-miR-1247-3p	chr 12	301.0	nd
mmu-miR-3474	chr 2	24.6	nd
mmu-miR-192-3p	chr 19	23.2	nd
mmu-miR-1258-5p	chr 18	22.9	nd
mmu-let-7e-3p	chr 17	18.7	nd
mmu-miR-137-3p	chr 3	14.5	NIX, involved in mitochondrial autophagy [67] MEF2A, involved in mitochondrial dynamics [68] FUNDC1, NIX, involved in mitophagy [69]
mmu-miR-1193-5p	chr 12	13.6	nd
mmu-miR-292b-5p	chr 7	12.0	nd
mmu-miR-3105-3p	chr 7	10.7	nd

nd, not yet determined

**Table 4:** Testis-specific top-10 mitomiRs according to their reads count.

miRNA name	chromosome location	reads count	Validated target related to mitochondria functioning [reference]
mmu-miR-465a-3p	chr X	2664.2	nd
mmu-miR-465b-3p	chr X	2664.2	nd
mmu-miR-465c-3p	chr X	2650.4	nd
mmu-miR-743b-3p	chr X	2188.7	nd
mmu-miR-465b-5p	chr X	1972.7	nd
mmu-miR-449a-5p	chr 13	1130.3	Myc, related to mitochondrial dysfunction [70]
mmu-miR-871-5p	chr X	890.4	nd
mmu-miR-878-3p	chr X	658.4	nd
mmu-miR-471-5p	chr X	642	nd
mmu-miR-298-5p	chr 2	497	Nox1, involved in ROS production and mitochondrial injury [71]

nd, not yet determined

**Table 5:** WAT-specific top-10 mitomiRs according to their reads count.

miRNA name	chromosome location	reads count	Validated target related to mitochondria functioning [reference]
mmu-miR-216b-5p	chr 11	94.1	nd
mmu-miR-142a-3p	chr 11	74.2	nd
mmu-miR-7654-5p	chr 8	62.3	nd
mmu-miR-6969-5p	chr 17	33.6	nd
mmu-miR-7023-5p	chr 4	22.6	nd
mmu-miR-193b-5p	chr 16	14.0	NFYA, involved in ROS mitochondrial production [72]
mmu-miR-410-3p	chr 12	13.1	HMGB1, involved in mitophagy [73]
mmu-miR-200a-5p	chr 4	10.0	nd
mmu-miR-500-3p	chr X	7.8	nd
mmu-miR-215-3p	chr 1	7.0	nd

nd, not yet determined

**Fig 5:**
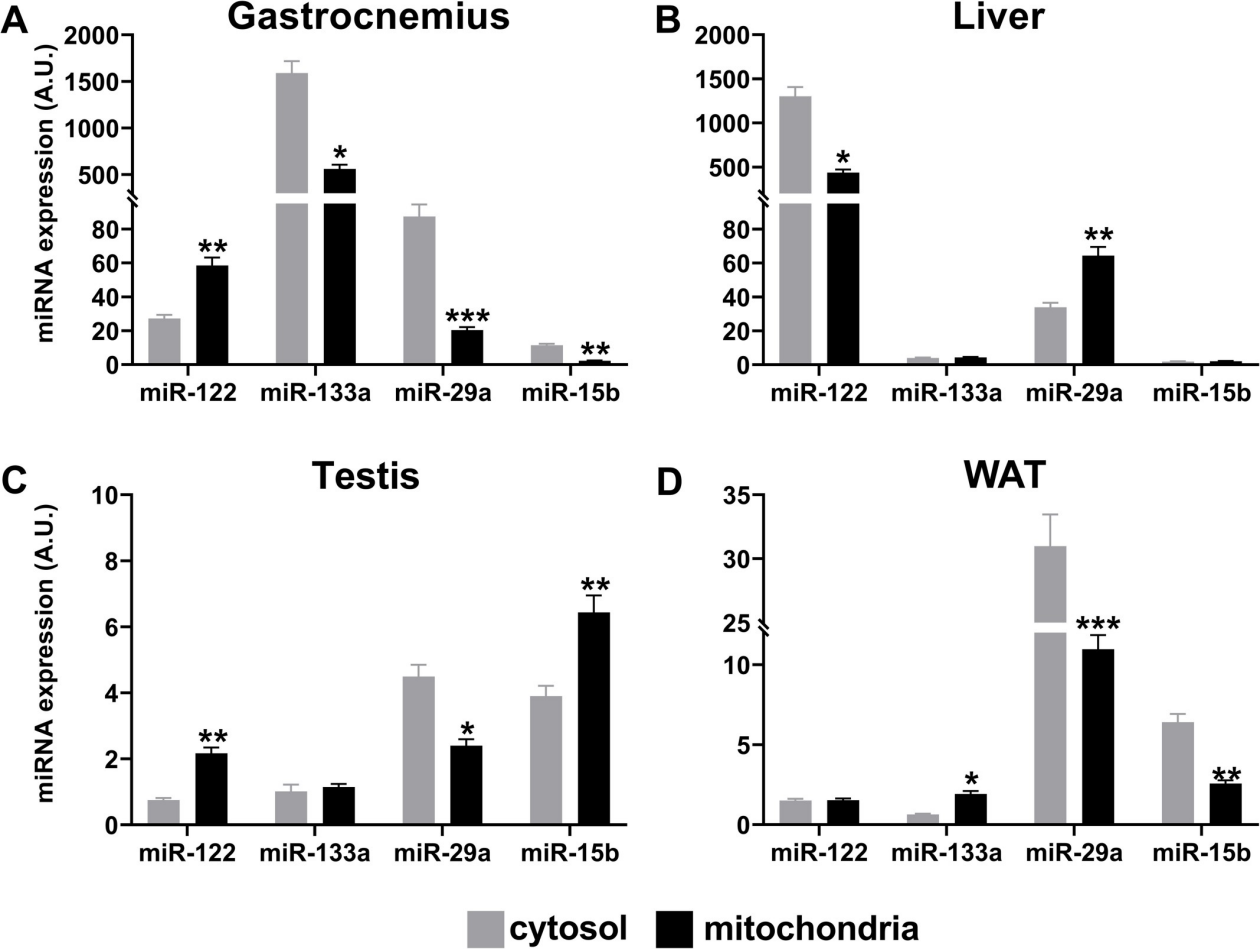
Expression patterns of miRNAs in cytosol and mitochondria fractions from the different tissues. Expression analyses of the indicated miRNAs were performed by using Taqman assays on the RNA extracted from cytosol and mitochondria fractions of the indicated tissues. All analyses were performed at least in triplicate on each sample and mean + sd is reported. p-values at Student’s t-test were *p < 0.05, **p < 0.01, or ***p < 0.001.

It would be interesting to address the question whether miRNAs found in mitochondria reflect the general content of the cells or are differentially distributed between cytosol and mitochondria. To gain some insights into the issue, we quantify the level of specific miRNAs in cytosol and mitochondria fractions; in particular, we analyzed the level of the 1^st^ and the 10^th^ of tissues-shared top-10 mitomiRs according to their reads count mean, i.e. miR-122-5p and miR-133a-3p (Table 1), and two other miRNAs, miR-29a and miR-15b, respectively 12- and 17-fold less expressed in comparison to miR-133a (S2 Table). We found that in most cases microRNAs are differentially distributed between cytosol and mitochondria, appearing significantly enriched in mitochondria, (e.g., miR-122 in gastrocnemius, miR-29a in liver, miR-122 and miR-15b in testis, miR-133a in WAT), or in lower amount in comparison to the cytosol (e.g., miR-133a, miR-29a and miR-15b in gastrocnemius, miR-122 in liver, miR-29a in testis and WAT, miR-15b in WAT), pointing to a potential relevance of selective translocation into the organelles (Fig 5).

### mitomiR targetomes and pathways

miRNAs exert their function by down-regulating multiple target transcripts, since their pairing can tolerate mismatches, beyond the perfect complementarity required for miRNA seed sequence (nucleotides 2–8). By screening the published papers on PubMed (searching keywords: “miRNA name AND mitochondria”), we found that various mitomiRs detected in our analyses have experimentally validated targets related to mitochondria function (Tables 1–5); however, gaps in the knowledge emerge for many others. To gain insights into biological functions of identified mitomiRs, the whole targetomes of all tissue-shared and all tissue-specific mitomiRs and related biological pathways were analyzed. In brief, we obtained a list of 4840 non-redundant transcripts experimentally validated or predicted by both TargetScan and miRDB tools as targets of the 126 tissue-shared mitomiRs; a list of 705 non redundant transcripts for the 21 mitomiR specifically found in gastrocnemius muscle; a list of 632 for the 30 mitomiRs for liver; a list of 4897 for the 174 testis-specific mitomiRs; a list of 520 for 18 WAT-specific mitomiRs.

**Fig 6:**
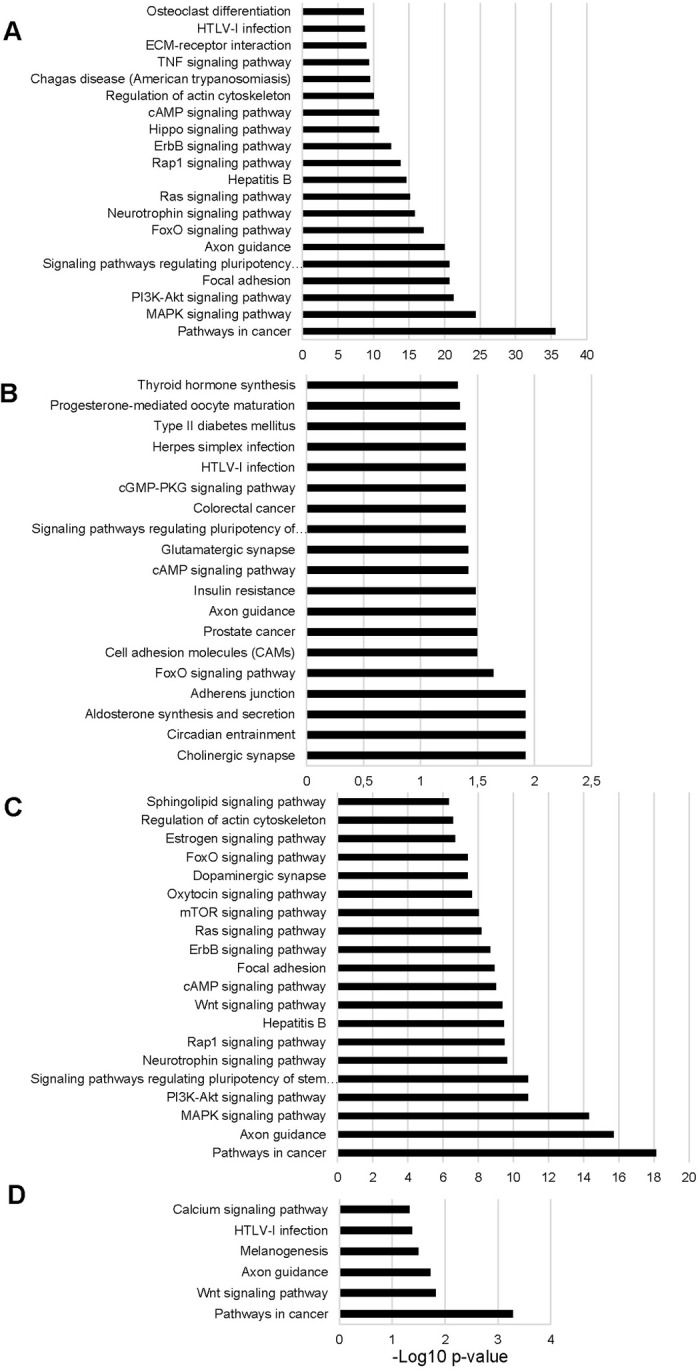
Biological pathways potentially governed by tissue-shared and tissue-specific mitomiR targetomes. The whole targetomes (predicted and validated) of tissue-shared or tissue-specific mitomiRs were analyzed by the program DAVID. Significantly enriched pathways according to KEGG are showed for tissue-shared (A; top-20 p values), liver-specific (B), testis-specific (top-20 p values) and WAT-specific mitomiRs; “PI3K-Akt signaling pathway” was the only one significantly enriched for gastrocnemius-specific mitomiRs targetome; full lists are given as Supplementary material. Biological pathways were considered statistically significant if p value was less than 0.05 (Benjamini–Hochberg procedure for multiple correction).

Enrichment of biological pathways supplied by KEGG on the targetome of tissue-shared mitomiRs shows an overrepresentation of different signaling pathways among the top p-values scored, beyond “Pathways in cancer”, the most studied field in relation to microRNAs (Fig 6 and S3 Table). That observation is also applicable on KEGG analysis performed on targetome of testis specific mitomiRs, although including some different signaling pathways. The enrichment of biological pathways implied in different signaling routes was probably expected for miRNAs associated to mitochondria, since the central role of the organelle in metabolism and homeostasis and the results here reported highlight the potential strong miRNA contribution. Behind those pathways enriched for mitomiRs arising from all analyzed tissues, others are specifically found for tissue-specific mitomiRs; e.g, “insuline resistance” and “Type II diabetes mellitus” pathways were found for liver-specific mitomiRs; “Wnt signling pathway”, well-known to be related to gluconeogenesis, was found for WAT; “Regulation of actin cytoskeleton” pathway emerges significantly enriched for mitomiRs in testis, where actin remodeling contributes to proper spermatogenesis in terms of morphology and then motility [74]. Overall, those results support the idea that regulation via miRNAs could be functionally relevant for mitochondria biology, since a pool of miRNAs could be related to the basic function of mitochondria shared by different cell types, and others may be related to the specialized functions of each tissue.

**Fig 7:**
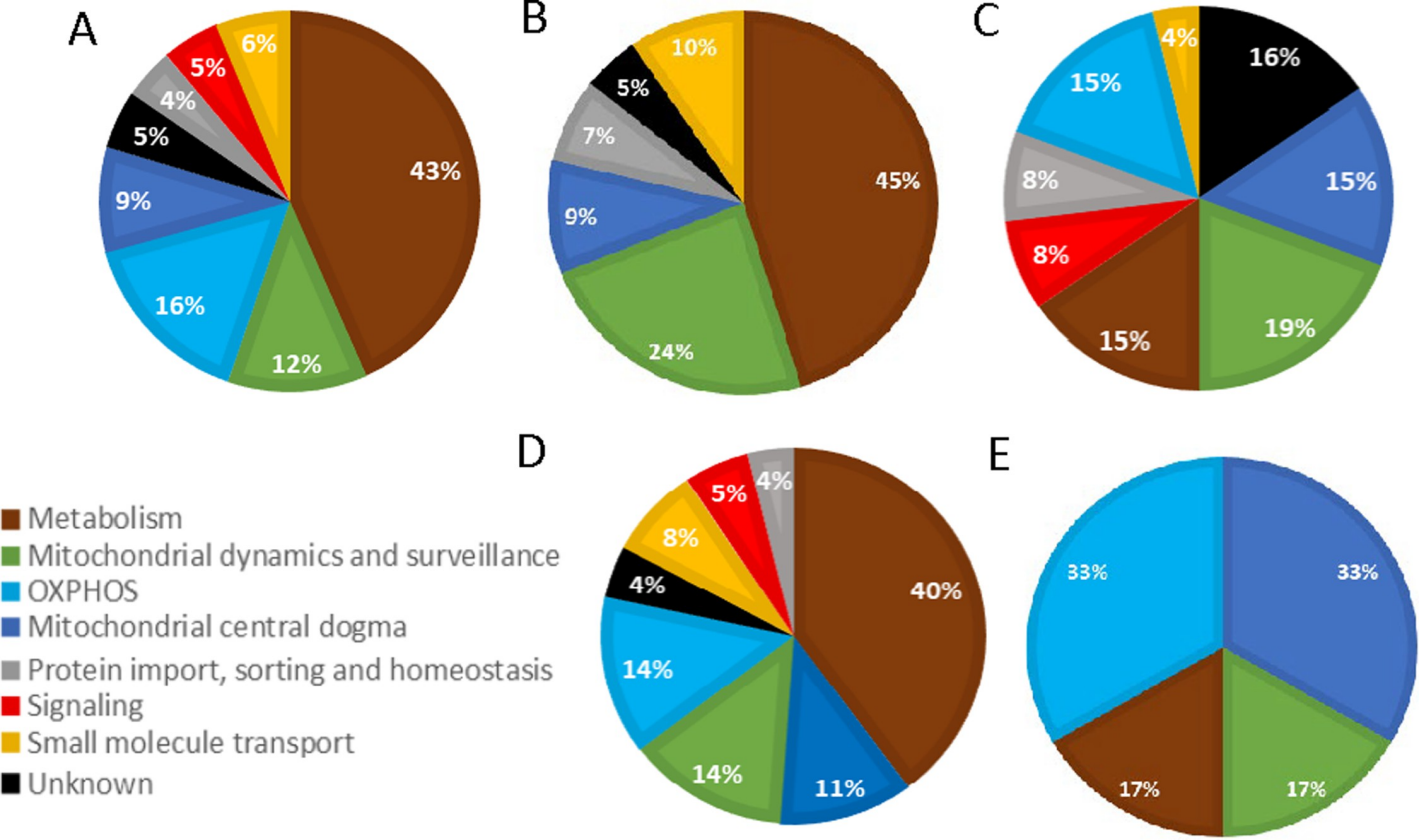
Mitochondrial-related biological pathways potentially governed by tissue-shared and tissue-specific mitomiRs. The targetomes of tissue-shared (A) and tissue-specific mitomiRs (B, gastrocnemius muscle; C, liver; D, testis; E, WAT) were further processed as described in Materials and Methods and then analyzed by MitoCarta3.0 for assignment to the seven broad functional categories relevant to mitochondria (“MitoPathways”).

KEGG annotations was helpful to put mitochondrial pathways potentially governed by miRNAs in a larger context, but we were also interested in exploring mitochondria-centric pathway annotations complementary to the broader pathway database. To that aim, the targetome lists were matched with 1140 transcripts encoding proteins with mitochondrial localization in the database of MitoCarta3.0, manually curated by adding experimentally validated transcripts targets as resulting from PubMed for our detected mitomiRs (Fig 7 and S4 Table). The assignment to the “MitoPathways” spanning the 7 categories related to mitochondria showed that they are all represented for tissue-shared mitomiRs targetome, with an overrepresentation of “Metabolism”, thus indicating a miRNA-mediated regulation contributing to the key roles of mitochondria. “Metabolism” is also prevailing on the 7 categories emerging from testis-specific mitomiRs targetome and on the 5 categories represented for gastrocnemius muscle. “Mitochondrial dynamic and surveillance”, “Metabolism”, “Oxphos” and “Mitochondrial central dogma” have prevailing and similar enrichment percentage for targetome of liver-specific mitomiRs, but also all the other categories are represented. With regard to WAT-specific mitomiRs targetome, 4 categories appeared enriched, with “OXPHOS” and “Mitochondrial central dogma” as the prevailing ones. These analyses point to a role of mitomiRs in regulating the different mitochondria-related pathways, but also show gaps in the knowledge (e.g, 16% of unknown pathways for liver) that should be filled with future experimentation.

Overall, targetome analyses of detected mitomiRs indicated that they can strongly support mitochondria biology and their regulatory power should be further investigated in a mitochondria-centric view, given the key role of the organelle in physiology and pathology.

## Discussion

Mitochondria are “multitasked” organelles, tailoring even their morphology, number and distribution to execute specialized functions in different cell types and/or different environments, behind the oxidative phosphorylation. Proteins and protein-driven pathways involved in mitochondria homeostasis are well known, whereas non-coding RNA contribution is still overlooked. In order to gain insights into possible new and specialized function of small RNAs related to mitochondria biology, here we performed a survey of small RNA content of mitochondria purified from different tissues that particularly relies on mitochondria functioning (Fig 1).

It is described for the first time the presence of mitopiRNAs in somatic tissues, behind testis tissues, thus supporting the idea of a strong and ubiquitous connection between mitochondria biology and piRNA pathways (Figs 2 and 3). piRNAs represents an emerging and fascinating class of short RNA, longer than miRNAs, usually 24–35 nt, whose biogenesis and functioning still require further investigation along with validation of those piRNAs identified only on their size, rather than on their PIWI-interaction or functional roles. It is well established that piRNAs are enriched in germ cells, where they modulate repression of transposable elements, and probably also gene expression regulation [5–7]. With regard to biogenesis, piRNAs are generally transcribed as longer precursors from specific genomic regions termed “piRNA clusters”; then, they are processed into mature piRNAs trough two interconnected pathways, ping-pong amplification mediated by PIWI proteins in ribonucleoprotein granules, and phasing, where PIWI-loaded piRNA precursors are translocated to the mitochondrial outer membrane by RNA helicases and further cleaved by endonuclease Zucchini/PLD [75]. Also PIWIL-1 was observed to localize to mitochondria, thus raising the possibility of a mitochondrial role in the biogenesis of piRNAs [44]. piRNAs in mitochondria have been already reported, but only for mammalian cancer cells [22, 44] and inferred by bioinformatic analyses on RNA purified from the whole cell lysates deriving from murine primordial germ cells, spermatozoa, oocytes, zygotes and embryonic gonadal cells [21, 41]. Those studies also suggest the possibility that some piRNAs may be generated also from mitochondrial genome. In the mitochondria purified from the tissues, we found different populations of piRNAs, shared or tissue-specific, but none of them seems to be produced by mitochondrial genome. To assess that, all piRNA sequences contained in piRBAse were aligned to mouse mitochondrial genome, finding 7.29% of perfectly matching piRNA full sequences (S5 Table), and thus possibly deriving from mitochondrial genome, consistently with data already reported [41]; however, no reads related to those perfectly matching piRNAs were found in our samples. Different reads related to the other piRNAs, but not completely covering their full sequences, cross-map to mitochondrial genome (S6 Table and S1 Fig), probably due to the presence of NUMTs, nuclear- mitochondrial identical DNA sequences, found in many metazoans [14, 76]. Overall, our data indicate that mitopiRNAs populations found here and commonly or specifically expressed in the different tissues are generated by nuclear genome, translocated to mitochondria and involved in a piRNA-mediated communication between mitochondria and nucleus, whose mechanisms and biological meaning deserves further investigation.

With regard to miRNAs, it is known that they can regulate different pathways related to mitochondria, e.g. by targeting transcripts encoding proteins involved in TCA cycle, OXPHOS, and mitochondrial dynamics [13, 75]. Although miRNAs are largely operative in the cytoplasm, some other papers report their localization in the nucleus and mitochondria [13, 15–22], whose significance and mechanism of action are still largely unknown, since the debated presence of RISC components inside the organelle. Here we describe a mitomiRs landscape, finding a group of mitomiRs shared by all tissues analyzed, and thus potentially endowed with a regulatory role required for general mitochondria functioning, and tissue-specific mitomiRs, potentially related to specialized functions dependent on cell context (Fig 4). Among the top-10 expressed miRNAs, different ones have experimentally validated targets related to mitochondria functioning, as reported in Tables 1–5; however, for many of them studies demonstrating possible functional links with mitochondria are still missing and could be inspired by their finding inside the organelle. To gain insights into biological functions of detected mitomiRs, we extended our analyses to the whole targetomes of all tissue-shared and all tissue-specific mitomiRs, considering both experimentally validated and predicted targets and their enrichment in biological pathways. KEGG analyses highlighted that the tissue-shared mitomiRs greatly contribute to the central role of mitochondria in different signaling routes, overrepresented among the top p-values scored pathways; mitomiRs detected in specific tissues potentially contribute to regulate biological pathways typical of those tissues, as detailed in Results, overall supporting the idea of a shared population of mitomiRs for basic functioning and others for specialized functions related to the different tissues. In a more mitochondria-centric view, exploitation of MitoCarta3.0 database further confirm the presence of targets encoding proteins related to mitochondria functioning in the whole targetomes and their involvement in pathways specific of the organelle such as “Metabolism” and “OXPHOS” (Fig 7 and S4 Table).

With regard to mapping of mitomiRs to nuclear genome, intriguingly, 8 out of 10 most expressed mitomiRs specifically found in testis is produced by X chromosome (Table 5), consistently with data already reported and arguing a special role of miRNAs from X chromosome in male fertility [77, 78].

Finally, it is debated if mitomiRs could also derive from mitochondrial genome, although the mainstream view is that they originate from the nucleus and then translocated to mitochondria as illlustrated in Introduction. In order to give our contribution to the debate, we followed the same workflow described above for piRNAs to assess the miRNAs origin in our sample: all murine miRNAs deposited in miRbase were aligned to mitochondrial genome, finding only mmu-miR-6481 and mmu-miR-12206-5p sequences perfectly matching as full sequences to mitochondrial genome. However, those 2 miRNAs are not represented in our RNAseq data, instead finding reads related to other miRNAs, although not covering their full sequences (S6 Table and S1 Fig). Also taking into consideration the absence of Dicer in mitochondria [18, 47, 79], the data suggest that in our samples the reads related to miRNAs and mapping to mitochondrial genome can be attributed to miRNAs produced by their well-known chromosomal location, and could cross-map to mitochondrial genome for the presence of NUMTs, as also discussed above for mitopiRNAs [14, 76]. Besides miRNAs and piRNAs, other small non-coding RNA biotypes (generally called mitosRNA) could be produced by mitochondrial genome, not affected by Dicer absence, but probably products of currently unidentified mitochondrial ribonucleases, and whose biogenesis and role still remain unknown requiring further investigation [47].

Overall, our analyses represent a large data platform obtained by RNAseq on highly purified mitochondria from different tissues, suggesting the idea of a “basic” small RNA population residing into mitochondria and shared by different tissues, and others restricted to specific tissues. The significantly different profiles between mitochondria and cytosol fractions for some analyzed mitomiRs (Fig 5) support the relevance of miRNAs for mitochondria functioning and a potential selective translocation. The next challenge will be the planning of functional studies to deepen our understanding of small RNA processing, the shuttling among different subcellular compartments and their governed regulatory networks contributing to mitochondria biology.

## Supporting information

S1 Table mitopiRNAs detected in the current study, as annotated in RNA central (4 excel sheets). (XLSX)

S2 Table Full lists of tissues-shared and tissue-specific mitomiRs detected in the current study (5 excel sheets). (XLSX)

S3 Table Significantly enriched pathways according to KEGG of tissues-shared and testis-specific mitomiR targetomes (2 excel sheets). (XLSX)

S4 Table mitomiR targetome enrichments for the 149 MitoPathaways, as analyzed by MitoCarta3.0 (5 excel sheets). (XLSX)

S5 Table piRNAs from piRBase aligned to mitochondrial genome. (XLSX)

S6 Table piRNA and miRNA related reads aligned to mitochondrial genome, found in the different tissues and graphically reported in S1 Fig (8 excel sheets). (XLSX)

S1 Fig Mapping of piRNA and miRNA related reads to murine mitochondrial genome. Starting from the inner space, the four green tracks report reads (represented by radial bars) related to piRNAs found in gastrocnemius muscle, liver, testis and WAT tissues; then, the four blue tracks report reads related to miRNAs found in the same tissues with the same order as above. The red circle represents the mitochondrial genome (NC_005089) 16299 bp long and its genes with position indicated by numbers on external circle. Full information is included in S6 Table.
(TIFF)
