# Supplementary figures and images for: A landscape of mouse mitochondrial small non-coding RNAs
Source: PLoS One. 2024 Jan 2;19(1):e0293644. doi: 10.1371/journal.pone.0293644 (PMC10760717; doi:10.1371/journal.pone.0293644)

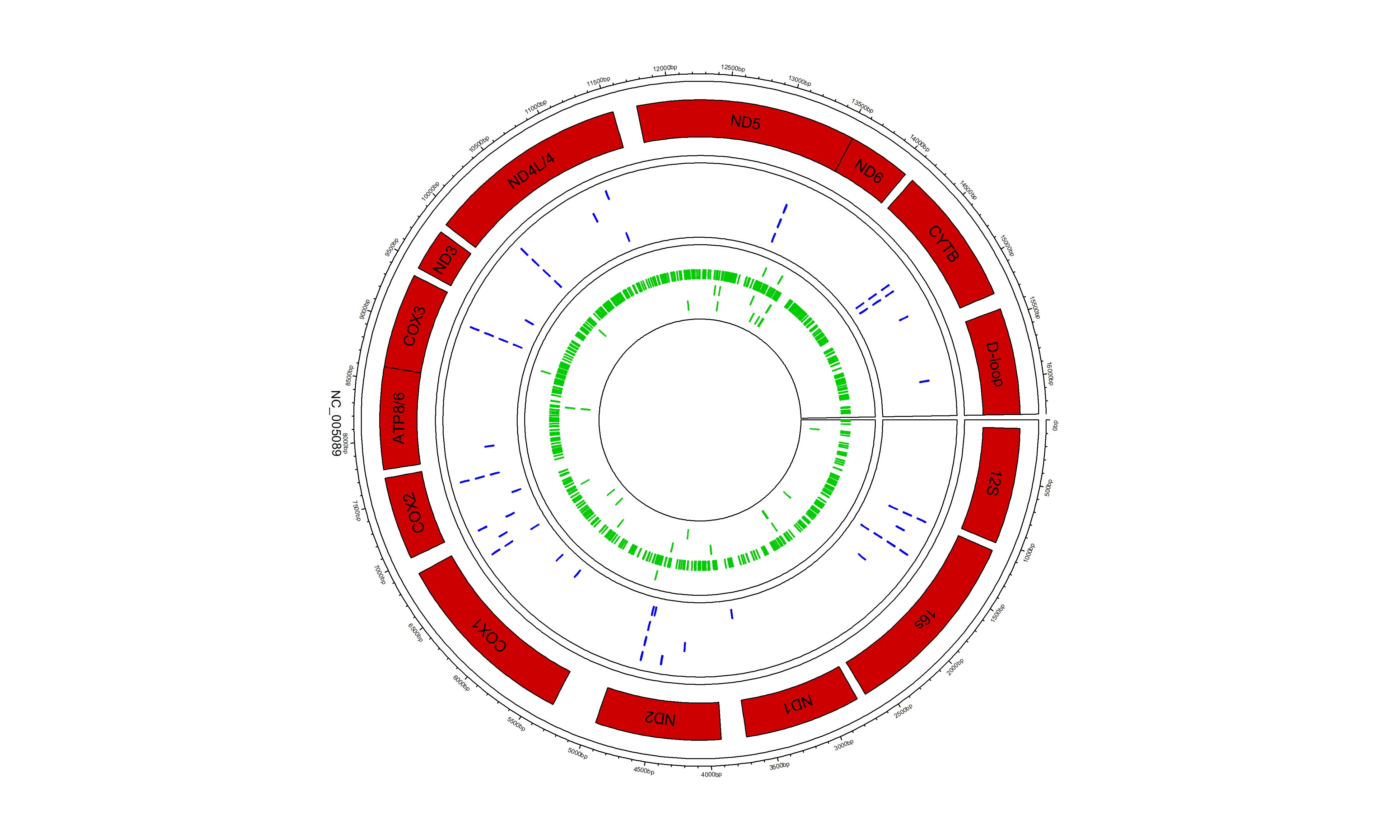

Supplement: S1 Fig — Starting from the inner space, the four green tracks report reads (represented by radial bars) related to piRNAs found in gastrocnemius muscle, liver, testis and WAT tissues; then, the four blue tracks report reads related to miRNAs found in the same tissues with the same order as above. The red circle represents the mitochondrial genome (NC_005089) 16299 bp long and its genes with position indicated by numbers on external circle. Full information is included in S6 Table. (TIFF) [file pone.0293644.s007.tiff]
